# Biomarkers of Exposure: A Case Study with Inorganic Arsenic

**DOI:** 10.1289/ehp.9058

**Published:** 2006-06-12

**Authors:** Michael F. Hughes

**Affiliations:** National Health and Environmental Effects Research Laboratory, Office of Research and Development, U.S. Environmental Protection Agency, Research Triangle Park, North Carolina, USA

**Keywords:** arsenic, biomarker, biomonitoring, exposure, risk assessment

## Abstract

The environmental contaminant inorganic arsenic (iAs) is a human toxicant and carcinogen. Most mammals metabolize iAs by reducing it to trivalency, followed by oxidative methylation to pentavalency. iAs and its methylated metabolites are primarily excreted in urine within 4–5 days by most species and have a relatively low rate of bioaccumulation. Intra- and interindividual differences in the methylation of iAs may affect the adverse health effects of arsenic. Both inorganic and organic trivalent arsenicals are more potent toxicants than pentavalent forms. Several mechanisms of action have been proposed for arsenic-induced toxicity, but a scientific consensus has not been achieved. Biomarkers of exposure may be used to quantify exposure to iAs. The most common biomarker of exposure for iAs is the measurement of total urinary arsenic. However, consumption of seafood containing high concentrations of organic arsenic can confound estimation of iAs exposure. Because these organic species are thought to be relatively nontoxic, their presence in urine may not represent increased risk. Speciation of urinary arsenic into inorganic and organic forms, and even oxidation state, gives a more definitive indication of the exposure to iAs. Questions still remain, however, as to how reliably the measurement of urinary arsenic, either total or speciated, may predict arsenic concentrations at target tissues as well as how this measurement could be used to assess chronic exposures to iAs.

## Background

### Sources

Arsenic in the environment is from natural and anthropogenic sources. It is commonly bound to carbon, iron, oxygen, and sulfur, forming inorganic and organic arsenicals in various oxidation states. The physico-chemical properties of the many arsenic species are important determinants of their potential toxic effects.

#### Natural

Arsenic, primarily in its inorganic form, is ubiquitously found in soil, air, and water. More than 200 mineral species contain arsenic. Arsenic binds with iron and sulfur to form arsenopyrite. Background levels of arsenic in soil range from 1 to 40 mg/kg [World Health Organization (WHO) 2001]. Volcanic activity and soil microorganisms release arsenic into the air. Arsenic air levels vary throughout the world, with lower levels in rural areas (0.007–28 ng/m^3^) and higher levels in urban areas (3–200 ng/m^3^) (WHO 2001). Water dissolves minerals that may release arsenic. Inorganic arsenic (iAs) levels in seawater and fresh water range from 1 to 10 μg/L (WHO 2001), although levels 1,000 times greater have been recorded (Nordstrom 2002).

#### Anthropogenic

The major anthropogenic sources of iAs are nonferrous metal smelters and coal-burning energy producers. These processes contaminate air, water, and soil with iAs. Areas near nonferrous metal smelters may have air concentrations of arsenic > 1,000 ng/m^3^ (WHO 2001). The manufacture and use of arsenical pesticides and the improper handling of tailings from metal mining operations may contaminate surrounding environments.

### Uses

Many commercial, medical, veterinary, and pesticide products contain arsenic. Arsenic is used in the manufacture of semiconductors and glass and as a chemotherapeutic agent, pesticide, and growth promoter in farm animals (WHO 2001).

## Human Exposure to Arsenic

The diet provides the major amount of arsenic resulting from its nonoccupational human exposure. The estimated daily intake of arsenic in the United States ranges from 2 to 92 μg/day (Tao and Bolger 1998). Levels of iAs are highest in grains (74 ng/g) and produce (9 ng/g) (Schoof et al. 1999). For these foods, iAs constitutes 17–24% of total dietary arsenic. In contrast, organic arsenic (e.g., arsenobetaine) predominates in seafood. Some seafoods have levels of organic arsenic in the parts-per-million range (WHO 2001). Overall, organic arsenic appears to be the major form of dietary arsenic.

Regarding worldwide public health, the most important medium for iAs exposure is drinking water. The source of iAs in drinking water is primarily geologic (Nordstrom 2002). Approximately 98% of the U.S. population ingests drinking water containing < 10 μg As/L (Chappell et al. 1997). Chronic exposure to elevated levels of iAs in drinking water is associated with the development of cancer and other adverse outcomes (Table 1) [National Research Council (NRC) 1999, 2001; WHO 2001]. Millions of people worldwide ingest drinking water contaminated with iAs at levels > 100 μg/L (Chatterjee et al. 1995; Smith et al. 2000).

Occupational and nonoccupational exposure to iAs may occur by inhalation at or near nonferrous smelters, residential and industrial burning of coal, or pesticide manufacturing plants and by dermal contact of arsenic-contaminated soil or use of arsenic-containing pesticides. Estimates of arsenic air concentrations at a Tacoma, Washington, copper smelter exceeded 1,000 μg/m^3^ during certain periods of its operation (Enterline et al. 1987).

Wood containing the preservative chromated copper arsenate (CCA) is of recent interest. In the United States, CCA-treated wood is found in many residences and playgrounds. Approximately 5 times more water-soluble arsenic was found on the hands of children who played on playgrounds built with CCA-treated wood than of children who played on equipment without CCA-treated wood (Kwon et al. 2004). Due in part to the concern about exposure to children, the use of CCA-treated wood in homes and playgrounds was voluntarily phased out in 2003.

## Arsenic and Biomarkers

Biomarkers are classified as those of exposure, effect, and susceptibility. For arsenic, biomarkers of exposure have received the greatest attention. The most common arsenic biomarker of exposure is the analysis of total arsenic in urine. Urinary porphyrins have also been proposed as an arsenic biomarker of exposure (Wang et al. 2002). After chronic ingestion of iAs, dermatologic lesions may develop. These lesions have been used as a long-term biomarker of cumulative arsenic exposure (Chen et al. 2005). Candidate arsenic effect biomarkers include clastogenicity in peripheral lymphocytes (Maki-Paakkanen et al. 1998), micronuclei in oral mucosa and bladder cells (Biggs et al. 1997), and induction of heme oxygenase (Del Razo et al. 2001a). A potential susceptibility biomarker is variability in arsenic metabolism, which reflects polymorphisms in the genes that encode the arsenic-metabolizing enzymes (Vahter 2000).

## Disposition of Arsenic

iAs is readily absorbed after oral exposure by most mammalian species. Absorption of iAs after inhalation is relatively less than after oral exposure and is more limited after dermal exposure (NRC 1999; WHO 2001). After oral absorption, iAs is primarily methylated in the liver and excreted in urine by most species. The metabolic pathway (Equation 1) of iAs involves sequential two-electron reduction of As^V^ species [arsenate, iAsV; monomethylarsonic acid, MMA^V^; dimethylarsinic acid, DMA^V^) followed by oxidative methylation of As^III^ species (arsenite, iAs^III^; monomethylarsonous acid, MMA^III^; dimethylarsinous acid, DMA^III^) (Thomas et al. 2001):


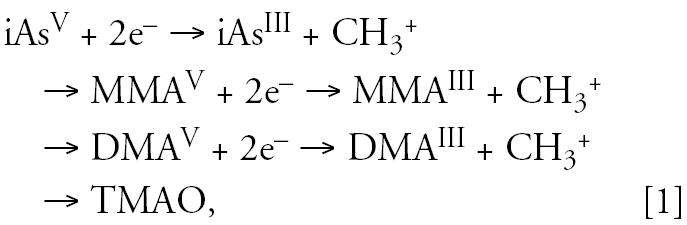


where TMAO is trimethylarsine oxide. The As^V^ species are reduced to As^III^ species *in vitro* nonenzymatically by thiols and enzymatically by purine nucleotide phosphorylase and MMA^V^ reductase (Aposhian et al. 2004; Thomas et al. 2001). The oxidative methylation of As^III^ species is an enzymatic process. Arsenic-methylating enzymes from rabbit and rat liver have been characterized. For *in vitro* activity, these enzymes require a thiol and the methyl donor *S*-adenosylmethione (Aposhian et al. 2004; Thomas et al. 2001). The rabbit enzyme appears to have two distinct methylating activities, one each for iAs^III^ and MMA^III^. The rat enzyme (AS3MT) has methyltransferase and reductase activities. Mice and humans have orthologues of the rat gene that encodes for AS3MT (Thomas et al. 2004). Regardless of the pathway of arsenic metabolism, exposure to iAs results in the urinary excretion of predominantly DMA^V^ and smaller amounts of inorganic and organic As^V^ and As^III^ species, including TMAO.

### Variation in arsenic metabolism

Metabolism of iAs varies between species and human populations (Loffredo et al. 2003; Vahter 2000; Vahter et al. 1995). Dogs and mice are rapid methylators of iAs and excrete ≥ 80% of the dose as DMA^V^ in urine. Humans excrete relatively more MMA^V^ than other species, suggesting that humans are slower methylators of iAs. This may explain in part why humans are more sensitive to iAs than other species. Arsenic excreted by humans tends to be 10–20% MMA^V^, whereas that of dogs, hamsters, mice, rabbits, and rats is 1–5% MMA^V^ (Vahter 2000). The guinea pig, marmoset monkey, and chimpanzee do not methylate iAs *in vivo* (Vahter 2000). The toxicologic effects of iAs in these animals are not known.

The distribution of arsenic in human urine is generally 10–30% iAs, 10–20% MMA^V^, and 60–70% DMA^V^ (Vahter 2000). Some populations excrete varying amounts of MMA^V^, both considerably less and more, in urine (Vahter 2000; Vahter et al. 1995). This suggests that there are genetic polymorphisms in the regulation of the enzyme(s) that metabolize arsenic, which may lead to differences in toxicity related to arsenic exposure. However, the intraindividual variation of iAs metabolism, measured over a 5-day period by Concha et al. (2002), appears to be stable over time.

## Biologically Active Agent

Methylation of iAs to DMA^V^ facilitates excretion of arsenic. Historically, DMA^V^ has been identified as being > 20-fold less acutely toxic than iAs, suggesting that methylation of iAs is a detoxication reaction. Improvements in analytical techniques have resulted in detection of MMA^III^ and DMA^III^ in the urine of individuals exposed to iAs (Aposhian et al. 2000; Del Razo et al. 2001b; Mandal et al. 2001). Thus, trivalent organic arsenicals are not the transitory intermediates previously believed, although their stability is an issue. MMA^III^ and DMA^III^ are potent *in vitro* and *in vivo* toxicants (Petrick et al. 2000, 2001; Styblo et al. 2000). DMA^V^, albeit at relatively high doses, is a multiorgan tumor promoter in rodents and a complete bladder carcinogen in rats (Wanibuchi et al. 2004). Methylation of iAs may not be a mechanism of detoxication but one of activation.

## Arsenic Toxicity

The toxic effects of arsenic are related to its oxidation state (Hughes 2002; WHO 2001); trivalent arsenicals are more potent than pentavalent arsenicals. The mechanism of action for arsenic toxicity is not clearly known. Trivalent arsenicals react directly with sulf-hydryls, a key component of many proteins. Arsenate, pentavalent iAs, has properties similar to those of phosphate (Dixon 1997). Arsenate may replace phosphate in critical biochemical processes that could lead to a toxic effect.

### Animal

The LD_50_ (median lethal dose) of iAs in rodents, depending on its oxidation state and route of administration, ranges from 10 to 90 mg/kg (WHO 2001). Arsenite is 3–4 times more potent than arsenate. MMA^III^ administered intraperitoneally is more acutely toxic than iAs^III^ in hamsters (Petrick et al. 2000). MMA^V^ and DMA^V^ are less potent than iAs in rodents, with LD50 values, depending on route of administration, ≥ 470 mg/kg (WHO 2001).

Adverse noncancerous effects of iAs include embryo and fetal toxicity, teratogenicity, genotoxicity (by indirect DNA- or chromosome-damaging mechanisms), and cardiovascular toxicity (WHO 2001).

### Human

The oral LD_50_ of iAs in humans is estimated to be 1–2 mg/kg (Ellenhorn 1997). Chronic exposure to iAs may result in cutaneous, developmental, hematologic, reproductive, and vascular effects (Table 1) [U.S. Agency for Toxic Substances and Disease Registry (ATSDR) 2000; NRC 1999; WHO 2001]. However, the potential for iAs to cause specific birth anomalies such as neural tube defects has been questioned (DeSesso et al. 1998).

The oral reference dose (RfD; a dose considered safe for regular daily consumption without adverse health effects) for iAs is 0.3 μg/kg/day, taking into account estimates of arsenic in food (2 μg As/day) and the volume of water consumed (4.5 L/day) [U.S. Environmental Protection Agency (U.S. EPA) 1993]. This RfD was based primarily on studies of a Taiwanese population, some of whom ingested high levels of iAs (400–600 μg/L) in drinking water (Tseng 1977; Tseng et al. 1968). A no observed adverse effect level of 0.8 μg/kg/day was derived from the critical effects of hyper-pigmentation, keratosis, and potential vascular complications (U.S. EPA 1993).

## Arsenic Carcinogenicity

The International Agency for Research on Cancer (1987) and the U.S. EPA (1993) have classified iAs, based on human evidence alone, as a group 1 and group A carcinogen, respectively. However, the mechanism of action for iAs-induced carcinogenicity is not known. Proposed mechanisms include genotoxicity, oxidative stress, inhibition of DNA repair, tumor promotion, cocarcinogenesis, cell proliferation, and altered signal transduction or DNA methylation (Hughes 2002; Kitchin 2001; Rossman 2003). More than one of these mechanisms may occur, and some may work together.

### Animal

The results of many cancer bioassays in which several species were administered iAs in the diet or drinking water or after oral intubation have been negative (Hughes 2002; Kitchin 2001; Rossman 2003). The lack of an animal model for iAs-induced carcinogenicity has hindered determination of a mechanism for this effect. However, recent studies with iAs have used transgenic mice (Chen 2000; Germolec et al. 1997), ultraviolet radiation as a co-carcinogen (Burns et al. 2004), and *in utero* exposure at relatively high doses (Waalkes et al. 2004). In these studies, tumors have developed in the iAs-exposed animals.

Rats administered high levels of DMA^V^ in the diet or drinking water developed bladder tumors (Arnold et al. 1999; Wei et al. 1999). DMA^V^ is also a multiorgan tumor promoter in rodents (Wanibuchi et al. 2004). Hepatocellular adenomas were significantly increased over background levels in rats administered TMAO in drinking water (Shen et al. 2003b). In contrast MMA^V^ administered in the diet (Arnold et al. 2003) or drinking water (Shen et al. 2003a) of rodents was not carcinogenic.

### Human

Lung cancer from occupational exposure to arsenic in smelter workers, miners, and pesticide manufacturers has been reported (NRC 1999; WHO 2001). Exposure to drinking water contaminated with iAs can lead to the development of cancer in skin, bladder, lung, kidney, and other internal organs (NRC 1999, 2001; WHO 2001).

Quantitative estimates for the risk of development of skin cancer from oral exposure to iAs are an oral slope factor of 1.5 mg/kg/day and a drinking water unit risk of 5 × 10^−5^ μg/L (U.S. EPA 1993). The arsenic inhalation unit risk for cancer, based on studies of occupational exposure to iAs, is 4.3 × 10^−3^ μg/m^3^ (U.S. EPA 1993).

A National Academy of Sciences committee analyzed the health effects of iAs in drinking water and reported theoretical maximum-likelihood estimates of excess lifetime risk of bladder and lung cancer in the U.S. population (NRC 2001). At 10 μg/L of arsenic in water, the incidence per 10^5^ people for bladder cancer was 12 for females and 23 for males. The incidence per 10^5^ people for lung cancer was 18 for females and 14 for males. These risk estimates are greater than those used by the U.S. EPA in its decision to lower the arsenic drinking water maximum contaminant level (MCL) from 50 to 10 μg As/L.

## Methodologies for Detection and Speciation of Arsenic

Many techniques are available to analyze arsenic in biological samples (B’Hymer and Caruso 2004; Francesconi and Kuehnelt 2004; Gong et al. 2002; Mandal et al. 2004; WHO, 2001). Methods to measure total arsenic include neutron activation, X-ray fluorescence, atomic absorption and fluorescence spectrometry, and inductively coupled plasma–atomic emission and –mass spectrometry. The latter two techniques are the most sensitive for total arsenic measurement (picogram range). Sample preparation can be burdensome for total arsenic measurement using the spectroscopic techniques. The organic arsenic in the matrix must be converted to iAs, usually by heating the sample to extreme temperatures in concentrated acid or by dry ashing.

Speciated analysis can differentiate inorganic from organic arsenic, and some techniques may maintain its oxidation state. Speciated analysis is performed by coupling chromatographic separation with a detector used for total arsenic analysis (Table 2). Sample preparation for speciated analysis is not as extreme as required for total arsenic analysis. In some cases, urine can be analyzed directly after removal of particulates by centrifugation.

The stability of arsenicals, particularly trivalent species, excreted in urine is a critical issue for speciated arsenic analysis. It is generally difficult to analyze urinary arsenic at a collection site. Storage of samples at 4 and −20°C appears to be suitable for maintaining the valence of some arsenicals for several months (Chen et al. 2002; Feldmann et al. 1999). Freeze-drying urine samples and storing frozen also extends stability of arsenicals (Feldmann et al. 1999; Yoshinaga et al. 2000).

Crecelius and Yager (1997) examined the variability in arsenic quantitation among several different laboratories. These laboratories analyzed standard solutions of MMA^V^ and DMA^V^, a reference sample and human urine spiked with iAs^III^, iAs^V^, MMA^V^, and DMA^V^. Different methods were used for total and speciated arsenic analysis. For samples that contained < 5 μg/L arsenic, the accuracy and precision were poor. However, the measurement of total iAs, MMA^V^, and DMA^V^ improved at levels > 5 μg/L, levels relevant to human exposure.

Standard reference material for arsenic is available in biological matrices such as urine, muscle, and liver but is certified only for total arsenic. Although a certified reference material for speciated arsenic in urine has been prepared (Yoshinaga et al. 2000), it is less readily obtainable. The identity of specific arsenicals has also become an issue. Hansen et al. (2004) reported that an arsenic sulfur compound that was detected in urine has been misidentified as DMA^III^. The availability of standard reference material for arsenic, which includes the trivalent methylated forms, has a tremendous impact on the ability to properly conduct an arsenic exposure analysis if biomarkers of exposure are to be used.

## Biomarker Characterization

Arsenic biomarkers of exposure include the analysis of urine, blood, hair, or nails for arsenic. Detection of arsenic in these biological samples is indicative of systemic absorption after exposure to it. However, arsenic from external sources may bind to hair and nails, which can complicate the exposure analysis.

### Urine

Absorbed arsenic is primarily excreted in urine, with a half-life of approximately 4 days in humans (NRC 1999; WHO 2001). Urinary arsenic is analyzed as total or speciated. Background levels of urinary arsenic range from 5 to 50 μg/L (NRC 1999). Excessive exposure to iAs in drinking water can lead to urinary arsenic levels > 700 μg/L (Buchet et al. 1999). For occupational exposure to iAs, the recommended biologic exposure determinant value is 35 μg As/L in urine (American Conference of Governmental Industrial Hygienists 2004). This level is a guideline for potential workplace health hazards and includes excreted iAs plus its methylated metabolites.

Quantitative correlations between the concentration of arsenic in urine and in air, water, or soil have been observed (Table 3). Linear relationships have been determined between urinary arsenic and iAs in water and soil. For iAs in soil, the correlations to urinary arsenic are less consistent; very high levels of iAs are generally necessary for a positive correlation to urinary arsenic (Valberg et al. 1997). This relationship is influenced by arsenic geochemistry and bioavailability of the arsenic in the soil matrix. For iAs in air, linear and nonlinear relationships have been reported with urinary arsenic. Pinto et al. (1976) reported a linear relationship up to 150 μg/m^3^ of arsenic, whereas Enterline et al. (1987) reported a nonlinear relationship with higher arsenic air concentrations. The nonlinear relationship was attributed to either the use of respirators by the workers at high arsenic air concentrations or changes in their arsenic storage or excretion mechanisms.

Certain foods contain organic arsenicals that can confound total urinary arsenic analysis. Some seafood contains high levels of arsenobetaine, an organic arsenical that is relatively nontoxic and excreted rapidly in urine intact (Vahter et al. 1983). Seaweed and marine algae contain arsenosugars that are metabolized to DMA^V^ after consumption and excreted in urine (Francesconi et al. 2002; Le et al. 1994; Ma and Le 1998). Aspects relating to the metabolism of arsenosugars have become more complex because of the recent identification of dimethylarsenic sulfur and acetate compounds in urine of sheep that ingested seaweed (Hansen et al. 2003, 2004). In an iAs exposure analysis for which seafood ingestion is suspected, speciation of urinary arsenic as well as determination of total urinary arsenic would be critical. If only total urinary arsenic was determined, exposure to iAs may be overestimated. For iAs exposure assessments, subjects should refrain from ingesting seafood 2–3 days before collection of urine.

Other concerns of urinary arsenic analysis are sample collection times (24 hr, spot, first morning void) and whether to adjust the data to volume of urine voided (related to urinary creatinine levels or specific gravity). Collection of urine for 24 hr can be difficult, because of quality assurance logistics, particularly if a large number of samples are to be collected. Urinary arsenic does not appear to vary over time, so spot collection or first morning void may be used (Calderon et al. 1999; Hewitt et al. 1995). In addition, intraindividual variation of iAs metabolism and its urinary excretion was low over a 5-day period (Concha et al. 2002). This suggests that methylation of arsenic may be steady over time by an individual who is continuously exposed to the same level of arsenic.

### Blood

Arsenic is cleared from blood within a few hours after it is absorbed (NRC 1999). Analysis of blood for arsenic is best suited for recent high-dose exposures. Background arsenic blood levels range from 0.5 to 2 μg/L (NRC 1999). Even though blood may attain steady-state levels after chronic exposure to iAs, it may not be a reliable biomarker of arsenic exposure because it is cleared so rapidly, particularly for low levels of iAs (ATSDR 2000).

A poor relationship exists between arsenic levels in drinking water and blood (WHO 2001). For levels of iAs in drinking water that ranged from 2.5 to 31 μg/L in a group of Andean women, blood arsenic levels were < 2 μg/L, and the total urinary arsenic concentration increased from 13 to 45 μg/L. In a group exposed to 200 μg/L iAs in water, blood arsenic levels increased to 8 μg/L, but total urinary arsenic increased to 261 μg/L (Vahter et al. 1995).

Blood is a more difficult matrix to work with than urine, and collection of it is an invasive procedure. Fewer subjects may participate in a blood collection study. Total arsenic analysis in blood may also present a problem if seafood was consumed by the subjects. Thus, if blood is to be sampled for determination of arsenic for exposure analysis, careful planning and a consistent sampling strategy should be employed.

### Hair and nails

Absorbed arsenic accumulates in hair and nails. This is thought to be due to the binding of As^III^ to sulfhydryl groups in keratin. Because hair and nails grow slowly, their analysis may give an indication of past arsenic exposure. Background arsenic levels in hair are < 1 μg/g (Hindmarsh 2002) and in nails range from < 1.5 to 7.7 μg/g (Agahian et al. 1990; Hinwood et al. 2003). iAs is the predominant form of arsenic in hair, with small amounts of dimethylated arsenic (Yamauchi et al. 1989). Based on animal studies, arsenobetaine does not accumulate in hair (Vahter et al. 1983), so consumption of seafood should not be a complicating factor in arsenic hair analysis.

Increased concentrations of arsenic in hair are observed in populations exposed to elevated levels of iAs in drinking water (Hinwood et al. 2003; Kurttio et al. 1998), air (Yamauchi et al. 1989), and soil (Hinwood et al. 2003). Kurttio et al. (1998) reported a significant correlation between arsenic in hair with total urinary arsenic (*r* = 0.75, *p* < 0.001), arsenic in drinking water (*r* = 0.74, *p* < 0.001), and daily dose of arsenic (*r* = 0.77, *p* < 0.001). With an increase of 10 μg/L of arsenic in drinking water, arsenic in hair increased 0.1 μg/g.

Populations exposed to elevated levels of iAs in drinking water (Hinwood et al. 2003), air (Agahian et al. 1990), and soil (Hinwood et al. 2003) show increased levels of arsenic in nails. Karagas et al. (1996) reported a significant correlation between detectable levels of arsenic in drinking water (> 1 μg/L) and in toenails (*r* = 0.83, *p* = 0.0001). With a 10-fold increase in arsenic in well water, the toenail arsenic concentration increased about 2-fold. In a follow-up study with a larger sample size, a significant correlation (*r* = 0.46, *p* < 0.001) between arsenic in water (0.002–66.6 μg/L) and nails (< 0.01–0.81 μg/g) was observed (Karagas et al. 2000).

The collection of hair and nails is not as invasive as collecting blood; more subjects may be willing to participate in iAs exposure studies if hair and nails are collected. A major issue in the use of hair and nails as biomarkers of exposure is their adsorption of arsenic from external sources (Hindmarsh 2002). For someone who consumes and bathes in water or is in contact with soil with elevated levels of iAs, arsenic from internal and external exposure would most likely be detected in hair and nails. This would complicate the exposure analysis. Although washing procedures have been developed, the possibility exists that this procedure may remove arsenic in the specimens that originated from internal sources. It is also not presently possible to distinguish between externally and internally derived arsenic in hair (Hindmarsh 2002).

## Exposure Assessment

Food and drinking water are the principal sources of nonoccupational exposure to iAs for most populations. The daily dietary intake of iAs for an adult in the United States ranges from 8 to 14 μg/day (Yost et al. 1998). Approximately 98% of the U.S. population ingests water containing < 10 μg As/L (Chappell et al. 1997). Thus, the nonoccupational exposure to iAs in most of the adult U.S. population (regular diet and 2 L of water/day) is < 50 μg/day. There are regions in the United States and the world where arsenic levels in drinking water are significantly elevated. If this water is regularly ingested, exposure to iAs would be significantly increased. Other sources of iAs exposure are soils at Superfund sites and where arsenical pesticides were produced or used. Wolz et al. (2003) reported on elevated levels of arsenic in and around homes constructed on or near fruit orchards in agricultural communities with historical use of lead arsenate. This study showed strong correlations between indoor and outdoor concentrations of arsenic and hence provided evidence for a “track-in” exposure pathway for residential environments. Occupational exposure of arsenic can occur at smelters, coal burning facilities, or arsenic pesticide manufacturing sites via inhalation. Populations that reside near these industries also have the potential for exposure to arsenic.

Walker and Griffin (1998) used an exposure assessment model (U.S. EPA 1989) to predict urinary arsenic concentrations of children living near a Superfund site. Using this model and site-specific data, the predicted urinary arsenic concentrations reasonably agreed with the measured urinary arsenic concentrations. The predicted risks from exposure to iAs using the site-specific data in the exposure model were less than those predicted risks using default values. The results of this study, which used biomonitoring data, show that current risk assessment approaches using default values can be conservative by overestimating risk.

### Evaluation of trends in exposure

Analysis of urine and blood is typically used for recent exposure to iAs. This is because arsenic is rapidly cleared from the blood and excreted in the urine after its systemic absorption (NRC 1999). Hair and nails may be used to assess past exposure to iAs. A major question in the use of urinary arsenic as a biomarker of exposure is how to relate the recent exposure, measured by urinary arsenic, to exposures that may have occurred chronically. Sporadic episodes of higher exposures within a chronic exposure context are also difficult to determine.

### Uniquely exposed populations

There are populations in India, Bangladesh, and other countries that are exposed to exceedingly high levels of iAs in their drinking water (Chatterjee et al. 1995; Nordstrom 2002). Children also represent a subgroup for unique exposure to iAs. Children differ from adults in that they are still developing and have dissimilar food and water consumption patterns and exposure to media, such as soil and CCA-treated wood, which may have a significant impact on the total exposure. Polissar et al. (1990) examined pathways of arsenic exposure in a population that resided near a copper smelter. These pathways included outdoor and indoor air, soil, and house dust. Elevated urinary arsenic levels were found primarily in children younger than 6 years who lived within 0.5 miles of the smelter. Hand-to-mouth activity appeared to be the main source of arsenic exposure to these children. *In utero* exposure to iAs, primarily from mothers consuming elevated iAs in drinking water, may represent another unique type of exposure. Knowledge of the effects of this type of exposure is limited, although elevated adverse pregnancy outcomes (e.g., stillbirths) have been reported in Bangladeshi women chronically exposed to iAs in drinking water (Ahmad et al. 2001).

## Effectiveness of an Intervention or Regulatory Action

The actions of governmental and nongovernmental organizations (Chowdhury 2004; Smith et al. 2000) that are attempting to reduce the drinking water exposure of iAs in the Bangladesh population are an example of the confirmed relationship of arsenic exposure, biomarkers, and health effects. Because of the relationship between drinking water exposure to iAs and development of cancer, the U.S. EPA lowered the arsenic drinking water MCL from 50 to 10 μg/L (U.S. EPA 2001). The U.S. Food and Drug Administration (2005) recently revised the bottled water quality standard at the same level. The WHO lowered its recommended arsenic drinking water guideline from 50 to 10 μg/L in 1993.

Approximately 2% of the U.S. population will be above the revised MCL for arsenic in 2006. One way to follow the effectiveness of this MCL would be to analyze urinary arsenic from a segment of this population after their arsenic drinking water levels are decreased. Hopenhayn-Rich et al. (1996) analyzed urine from a population previously exposed to high iAs (600 μg/L) drinking water levels and had switched to water containing less arsenic (45 μg/L). The average urinary arsenic levels in this population decreased from 636 to 166 μg As/L.

## Conclusions and Recommendations for Future Research

iAs is a ubiquitous environmental contaminant. Human exposure to it occurs from many types of media. Analytical techniques have been developed that can detect low levels of iAs in biologic samples. Based on the oral RfD and drinking water MCL, it appears that there are levels of iAs that most humans can be exposed to without the development of adverse outcomes. However, many questions about iAs still exist, and the recommendations listed below may aid in decreasing these uncertainties:

- Link biomarkers of arsenic exposure to effect.
- Standardize a method for arsenic species analysis in biological samples that ensures good quality assurance and control and considers stability issues after sample collection.
- Determine the mechanism of action for arsenic.
- Examine the intra- and interindividual variability of arsenic methylation and effect.
- Assess the efficacy of lowering the arsenic drinking water MCL from 50 to 10 μg/L.
- Understand the magnitude and duration of exposure to iAs.
- Develop physiologically based pharmacokinetic models for iAs low-dose extrapolation.
- Quantify the contribution of arsenosugars to dimethylarsenic levels in urine.
- Assess contribution of arsenic in diet to overall exposure.

Many of the studies referenced here have observed a significant and positive correlation between iAs in an exposure medium and arsenic in a biologic sample, principally urine, hair, and nails. With proper precautions and recognition of their limitations, analysis of these samples can be informative regarding whether an exposure to iAs occurred. It would be ideal if the biomarkers of exposure for iAs were linked to target tissue dose and, perhaps more important, to an adverse health effect in humans. For example, Enterline et al. (1987) reported a linear relationship between respiratory cancer mortality based on standardized mortality ratios and total urinary arsenic in smelter workers. More studies like Enterline et al. (1987) as well as in other areas are needed to develop a more robust risk assessment for iAs and enhance public health protection.

## Figures and Tables

**Table 1:** Range of chronic human oral exposures to iAs resulting in adverse effects.

System or effect	LOAEL (mg/kg/day)
Cardiovascular	0.002–0.067
Dermal	0.005–0.08
Endocrine	0.11
Gastrointestinal	0.015–0.06
Hematopoietic	0.05
Hepatic	0.006–0.1
Neurologic	0.005–0.11
Respiratory	0.015–0.08
Cancer	0.0011–3.67

LOAEL, lowest observable adverse effect level. Data adapted from ATSDR (2000).

**Table 2:** Examples of analytical techniques for speciation and detection of arsenic in urine.

Separation	Detection	Arsenic species	Level of detection	Reference
Cryogenic	Hydride generation–atomic absorption spectrometry	iAs^III^, iAs^V^, MMA^III^, MMA^V^, DMA^III^, DMA^V^, TMAO	0.14–0.4 ng	Devesa et al. 2004
HPLC (ion pair)	Hydride generation–atomic fluorescence spectrometry	iAs^III^, iAs^V^, MMA^III^, MMA^V^, DMA^III^, DMA^V^	10–40 pg	Le et al. 2000
HPLC (ion pair)	Hydride generation–inductively coupled plasma–atomic emission spectrometry	iAs^III^, iAs^V^, MMA^V^, DMA^V^	4–10 ng	Do et al. 2000
HPLC (anion exchange)	Hydride generation–inductively coupled plasma mass spectrometry	iAs^III^, iAs^V^, MMA^III^, MMA^V^, DMA^III^, DMA^V^, arsenocholine, arsenobetaine	3–7 pg	Mandal et al. 2004
HPLC (anion exchange)	Hydride generation–atomic absorption spectrometry	iAs^III^, iAs^V^, MMA^V^, DMA^V^, TMAO	0.11–0.26 ng	Sur and Dunemann 2004

HPLC, high-performance liquid chromatography.

**Table 3:** Quantitative relationships between the concentration of arsenic in exposure media and in urine or nails.

Exposure media	Biologic sample	Quantitative relationship	Reference
Air (3–295 μg As/m^3^)	Urine (μg/L)	*C*_air_ = 0.3*C*_urine_ (*p* < 0.01, *r* = 0.53)	Pinto et al. 1976
Air (50–3,500 μg As/m^3^)	Urine (μg/L)	*C*_air_ = 0.0064(*C*_urine_)^1.94^	Enterline et al. 1987
Air (< 0.1–35 μg As/m^3^)	Nails (μg/g)	*C*_air_ = 1.79*C*_nail_–5.9	Agahian et al. 1990
Water (8–620 μg As/L)	Urine (μg/mg creatinine)	10^−2.57^(*C*_water_)^0.63^ = log*C*_urine_ (*p* < 0.001, *r* = 0.655)	Calderon et al. 1999
Soil (102–356 μg As/g)	Urine (μg/L)	0.1955(log*C*_soil_) + 0.4818 = log*C*_urine_ (*p* < 0.001, *r* = 0.25)	Hwang et al. 1997
Soil (9–139 μg As/g)	Urine (μg/L)	3.025(*C*_soil_)^0.237^ = *C*_urine_ (*p* < 0.01, *r* = 0.21)	Ranft et al. 2003
